# Evaluation of MucorGenius® real-time polymerase chain reaction assay for mucormycosis diagnosis: a comparative analysis

**DOI:** 10.22034/cmm.2026.345408.1665

**Published:** 2026-02-01

**Authors:** Kanchan Ajbani, Siddhant Shetty, Saleha Chaudhry, Ekta Tatkare, Harshala Gaonkar, Shaoli Basu, Camilla Rodrigues, Anjali Shetty

**Affiliations:** Department of Microbiology, P D Hinduja hospital and Medical Research Centre, Mumbai, India

**Keywords:** Fungal infections, Molecular Diagnostics, Mucorales, Mucormycosis, Real-Time PCR

## Abstract

**Background and Purpose:**

Mucormycosis, a formidable invasive fungal infection linked to Mucorales fungi, poses a significant threat to individuals. Rapid initiation of suitable antifungal therapy within five days of diagnosis has shown a noteworthy reduction in mortality rates, emphasizing the critical importance of timely identification. However, the diagnostic landscape is marred by the limited sensitivity of routine tools, including direct examination, histology, and culture. In this context, this study aimed to scrutinize the diagnostic performance of the commercially available pan-mucorales real-time polymerase chain reaction (PCR) assay, MucorGenius® (Pathonostics), on both pulmonary and extra-pulmonary specimens. Its efficacy was compared with that of conventional mycology methods to address the pressing need for more accurate and timely mucormycosis diagnosis.

**Materials and Methods:**

In total, 27 clinical specimens were included in the study, 17 of which were obtained from patients clinically suspected to have invasive mucormycosis. The remaining 10 specimens were from patients with microbiologically confirmed pulmonary tuberculosis, who had no evidence of fungal infection; these served as non-fungal controls and were included specifically to assess assay specificity. The DNA extraction from all samples was performed using the Qiagen DNA minikit, and real-time PCR was conducted with the MucorGenius^®^ kit, targeting *Rhizomucor* spp., *Lichtheimia* spp., and *Mucor/Rhizopus* spp.

**Results:**

Of the 27 clinical cases, 17 were classified as either proven or probable mucormycosis based on EORTC/MSGERC criteria. The MucorGenius® PCR assay demonstrated an overall sensitivity of 76.47% and a specificity of 100% for detecting mucormycosis.

**Conclusion:**

The MucorGenius® Real-Time PCR assay exhibited promising diagnostic performance for mucormycosis, particularly in cases eluding conventional methods. Its sensitivity and specificity make it a valuable tool for early diagnosis, aiding clinical intervention in patients with a high index of suspicion.

## Introduction

Mucormycosis is a rare but aggressive fungal infection caused by *Mucorales* species, primarily affecting immunocompromised individuals, such as those with uncontrolled diabetes, hematologic malignancies, organ transplants, or prolonged corticosteroid therapy [1, 2]. It presents in various forms-rhinocerebral, pulmonary, gastrointestinal, cutaneous, and disseminated-each associated with high morbidity and mortality [3]. The COVID-19 pandemic contributed to a spike in cases, especially in patients receiving immunosuppressive therapy, emphasizing the need for early and accurate diagnosis [4].

Rhinocerebral mucormycosis, the most common form, typically occurs in patients with diabetes and ketoacidosis, presenting with nasal congestion, facial pain, periorbital swelling, and black eschars [5]. Without treatment, it can spread to the brain, causing life-threatening complications. Pulmonary mucormycosis, often seen in neutropenic patients, presents with fever, cough, and respiratory failure, with computerized tomography findings, such as halo or reverse halo signs, aiding diagnosis [6]. Gastrointestinal mucormycosis, though rare, is seen in malnourished individuals or neonates and often presents late with bowel ischemia or perforation [7]. Cutaneous mucormycosis may follow trauma or surgery and can rapidly invade deep tissues. Disseminated disease, affecting multiple organs, carries a grave prognosis [8].

Traditional diagnostic methods-direct microscopy, histopathology, and culture-have notable limitations. Microscopy lacks sensitivity and is operator-dependent [9]; histopathology is invasive and sometimes inconclusive [10]; and fungal culture, though a gold standard, is slow and often yields false negatives [11]. Additionally, serological tests, such as β-D-glucan and galactomannan, are ineffective in mucormycosis, as Mucorales lack these

Molecular diagnostics, particularly real-time polymerase chain reaction (PCR), offer rapid and sensitive detection of fungal DNA in clinical specimens [12]. The MucorGenius® assay (Pathonostics) targets key Mucorales genera (Rhizopus, Lichtheimia, Mucor) [13] and has shown greater sensitivity than culture, detecting fungal DNA in blood or tissue-sometimes before symptom onset.

This study aimed to evaluate the performance of the MucorGenius^®^ PCR assay on pulmonary and extra-pulmonary specimens from patients with suspected mucormycosis. Through comparison of its results to conventional methods, this study aimed to support the integration of advanced molecular diagnostics into routine care.

Given the growing burden of mucormycosis-driven by diabetes, immunosuppression, and post-COVID-19 complications- early diagnosis is critical for improving outcomes. Management involves prompt antifungal therapy (preferably liposomal amphotericin B), surgical debridement, and correction of underlying conditions [14]. In refractory cases, combination therapy with posaconazole or isavuconazole may be used. Incorporation of rapid molecular diagnostics, like MucorGenius®, could significantly enhance early detection and treatment, ultimately improving survival.

## Materials and methods

### Study design and ethical considerations

This was a retrospective diagnostic accuracy study conducted at a tertiary care academic hospital and national reference center for fungal diagnostics. The primary objective was to evaluate the diagnostic performance of the commercially available MucorGenius® real-time PCR assay (PathoNostics, Netherlands) for the detection of Mucorales DNA across various clinical specimens obtained from patients with suspected invasive mucormycosis. The study was conducted on de-identified archived specimens and formed part of an Indian Council of Medical Research-supported fungal laboratory for the development and validation of new tests for the identification of pathogenic fungi. As this was a retrospective study, stored leftover samples were used, for which conventional examinations-namely, clinical culture, microscopy, or histopathology-had already been performed as part of routine diagnostic work-up during initial patient care.

### Ethics Committee code

1272-19-AS

### Patient classification and case definitions

In total, 27 clinical samples were selected based on availability and completeness of microbiological workup. Cases were categorized according to the revised European Organization for Research and Treatment of Cancer/Mycoses Study Group Education and Research Consortium (EORTC/MSGERC) 2020 definitions [15] as follows:

- In this cohort of 17 mucormycosis-suspect cases, 8 (47%) fulfilled strict EORTC/MSGERC-2020 criteria for proven mucormycosis based on histopathologic, microscopy, or culture confirmation from sterile or deep-tissue specimens. The remaining 9 (53%) cases were classified as probable based on clinical features and culture from non-sterile or less definitive specimens.
- In the negative-control cohort (n=10), all patients had microbiologically confirmed pulmonary tuberculosis (TB) and no clinical, radiological, or microbiological evidence of fungal disease.

The goal was to capture a balanced cohort representative of real-world diagnostic challenges, particularly for specimens with low fungal burden or those affected by prior antifungal therapy.

### Specimen collection and handling

The 27 specimens comprised tissue biopsies (n=10) from sinus, bone, maxilla, and lower limb; bronchoalveolar lavage (BAL) fluid (n=5); sputum samples (n=5); pleural fluid or pleural tissue (n=3); and pus samples (n=4) from suspected soft tissue involvement.

All specimens were collected aseptically as part of routine diagnostic care and stored at 2–8 °C. After completion of standard testing, leftover material was aliquoted and stored at –20 °C to preserve nucleic acid integrity for PCR testing. Only samples with sufficient volume and metadata were included in the analysis.

### Conventional mycological diagnostics

Each specimen underwent direct microscopy using 10% KOH wet mounts and Calcofluor white staining to detect broad, aseptate hyphae; Lactophenol cotton blue (LPCB) staining for structural detail when fungal growth was achieved; and fungal culture on Sabouraud Dextrose Agar with and without chloramphenicol, incubated at 30 °C and 37 °C for up to 21 days.

Positive cultures were identified based on macroscopic colony morphology and microscopic features, including sporangia, sporangiophores, and rhizoids, using LPCB mounts. In cases of ambiguous morphology, subculture onto Potato Dextrose Agar was performed to enhance sporulation.

### Molecular diagnostics: MucorGenius® polymerase chain reaction assay

The DNA was extracted directly from the sample using the Qiagen QIAamp DNA Mini Kit following the protocol provided by the manufacturer. The extraction process involved enzymatic lysis with buffer AL and carrier RNA, ethanol precipitation, and DNA binding to silica columns, and Sequential wash steps and final elution in 100 µL of buffer AE.

Each sample was tested using the MucorGenius® real-time PCR assay, which targets conserved ribosomal DNA regions of clinically relevant Mucorales genera, including *Rhizopus*, *Mucor*, *Lichtheimia*, *Rhizomucor*, and *Cunninghamella*. The PCR reaction included an internal control (M13 bacteriophage DNA) to monitor for inhibition.

Amplification was performed on an ABI 7500 Fast real-time PCR system (Applied Biosystems, USA) based on the following thermal cycling conditions: initial denaturation at 95 °C for 3 min; 40 cycles of denaturation at 95 °C for 15 s and annealing/extension at 60 °C for 45 s. A cycle threshold value ≤ 35 was interpreted as positive for Mucorales DNA, in accordance with kit guidelines.

### Statistical analysis

Diagnostic accuracy was calculated using clinical case definitions as the reference standard. Key performance indicators included: sensitivity (true positives/all clinical positives); specificity (true negatives/all clinical negatives); positive predictive value (PPV) and negative predictive value (NPV); likelihood ratios (LR+and LR−) to quantify diagnostic confidence; receiver operating characteristic curve analysis to assess overall discriminative ability. All statistical calculations were performed in SPSS software (version 26.0, IBM Corp., Armonk, NY), with p < 0.05 considered statistically significant.

## Results

A total of 27 clinical specimens were analyzed in this study, which included 17 samples from patients with clinically suspected mucormycosis and 10 samples from patients with confirmed pulmonary TB who had no clinical, radiological, or microbiological evidence of fungal infection. The TB-positive patients served as negative controls to assess the specificity of the diagnostic assay. Based on the revised EORTC/MSGERC 2020 definitions for invasive fungal disease, patients with suspected mucormycosis were classified into proven or probable mucormycosis categories. Cases were adjudicated based on a combination of histopathology, microscopy, culture results, and clinical/radiological features.

This classification identified eight cases as proven mucormycosis, supported by histopathological evidence of Mucorales tissue or angioinvasion and/or positive fungal culture from sterile or deep-tissue samples, including pleural fluid, lung biopsies, and musculoskeletal tissue. The remaining nine cases were classified as probable mucormycosis, based on clinical and imaging findings as well as mycological evidence (microscopy and/or culture) obtained primarily from non-sterile specimens, such as BAL, sputum, or sinonasal biopsies without definitive histopathological evidence of tissue invasion. Table 1 summarizes this patient classification, showing the distribution of proven and probable cases alongside examples of sample types representative of each group.

**Table 1:** Patient classification by case category

Case Category	Number of Cases	Examples of Sample Types
Proven	8	Pleural fluid, lung biopsies, bone tissue, muscle tissue
Probable	9	Bronchoalveolar lavage, sputum, sinonasal tissues, mucosal biopsies

A total of 27 clinical specimens were analyzed in this study, including 17 samples from patients clinically suspected of invasive mucormycosis (classified as proven or probable) and 10 specimens from patients with microbiologically confirmed pulmonary TB, serving as non-fungal controls. All specimens underwent direct microscopy, fungal culture, and real-time PCR testing using the commercially available MucorGenius® assay. This PCR targets pan-Mucorales DNA with species-specific detection for *Rhizopus* spp., *Mucor* spp., *Rhizomucor* spp., and *Lichtheimia* spp.

Among the 17 mucormycosis-suspected patients, the MucorGenius® assay detected Mucorales DNA in 13 cases, yielding an overall sensitivity of 76.5% (13/17). All 10 TB control samples tested negative by PCR, microscopy/histopathology, and culture, resulting in a specificity of 100%.

When stratified by case category, PCR positivity was highest in proven mucormycosis cases (87.5%; 7/8), surpassing microscopy/histopathology positivity at 50.0% (4/8) and culture positivity at 62.5% (5/8). In probable cases (n=9), PCR positivity was 66.7% (6/9), compared to microscopy/histopathology positivity of 55.6% (5/9) and culture positivity of 22.2% (2/9) (Table 2).

**Table 2:** Diagnostic yield in the study cohort by exclusive positivity and polymerase chain reaction result

Case Group	Total (n)	Only Microscopy/Histopathology +, n (%)	Only Culture +, n (%)	Both Microscopy and Culture +, n (%)	PCR Positive, n (%)
Proven	8	2 (25.0%)	3 (37.5%)	3 (37.5%)	7 (87.5%)
Probable	9	5 (55.6%)	2 (22.2%)	2 (22.2%)	6 (66.7%)
Total	17	7 (41.2%)	5 (29.4%)	5 (29.4%)	13 (76.5%)
TB Controls	10	0 (0%)	0 (0%)	0 (0%)	0 (0%)

PCR: polymerase chain reaction, TB: tuberculosis

All 17 mucormycosis cases met the inclusion criteria requiring positivity by at least one classical mycological method (microscopy, histopathology, or culture). To enhance interpretability and avoid overlapping counts, classical mycology results were classified into three exclusive categories as follows: only microscopy/histopathology positive (positive by microscopy and/or histopathology but negative by culture), only culture positive (positive by culture but negative by microscopy/histopathology), and both microscopy/histopathology and culture positive. This stratification is detailed in Table 2 and illustrates diagnostic overlaps clearly.

Across all mucormycosis-suspected patients (n=17), microscopy/histopathology and culture demonstrated lower detection rates, with positivity in 52.9% (9/17) and 35.3% (6/17) of cases, respectively, highlighting the enhanced diagnostic sensitivity of the MucorGenius® PCR assay. In addition, the PPV, NPV, sensitivity, specificity, and overall accuracy of the MucorGenius® PCR assay were calculated against the EORTC/MSGERC classification of proven/probable mucormycosis cases (n=17) versus TB controls (n=10). The assay demonstrated a sensitivity of 76.47%, specificity of 100%, PPV of 100%, and NPV of 71.43%, with an overall diagnostic accuracy of 85.19%.

Two notable discrepant cases were observed during the study. The first involved a proven mucormycosis case in which the tissue biopsy grew *Apophysomyces* variabilis, whereas the corresponding clinical specimen tested negative by the MucorGenius® PCR assay. The PCR was performed on the culture isolate, which yielded a positive result. Similarly, a culture isolate of Saksenaea vasiformis was also tested, and a positive PCR result was observed. These findings suggest that while the assay may have some ability to detect rare species under controlled conditions, its performance on clinical specimens remains variable and may be influenced by fungal load or matrix effects. The second discrepant case involved a probable mucormycosis patient whose sputum culture grew Rhizopus arrhizus, but the clinical sample was PCR-negative. Potential explanations include low fungal DNA burden, PCR inhibitors, or DNA degradation. These cases underscore the importance of integrating PCR results with clinical, histopathological, and microbiological findings for accurate diagnosis.

When stratified by proven or probable case definitions, PCR positivity was higher among proven cases, with 87.5% (7/8) testing positive, compared to 66.7% (6/9) in probable cases. This observation aligns with the expectation that proven cases-often involving sterile site infections and higher fungal burdens-exhibit greater PCR sensitivity (Table 3).

**Table 3:** polymerase chain reaction positivity stratified by case category

Case Category	Number of Samples	PCR Positive, n (%)
Proven	8	7 (87.5%)
Probable	9	6 (66.7%)

PCR: polymerase chain reaction

## Discussion

Diagnosis of mucormycosis constitutes a clinical emergency due to the aggressive nature of this infection and the critical need for early intervention. However, diagnosis is frequently delayed owing to the inherent limitations of traditional modalities, such as direct microscopy and fungal culture. In this study, the commercially available MucorGenius® real-time PCR assay demonstrated markedly improved diagnostic performance, particularly in culture-negative cases and in specimens often challenging to evaluate, including pleural fluid and pus. These findings align with the emerging global evidence supporting the integration of molecular diagnostics into the routine workup of mucormycosis.

Although direct microscopy and fungal culture remain valuable first-line diagnostic tools, particularly when results are positive, their sensitivity is suboptimal. In the present study, the sensitivities of microscopy and fungal culture were 52.9% and 35.3%, respectively, in mucormycosis-suspected samples, compared to a substantially higher sensitivity of 76.5% achieved by the MucorGenius® PCR assay. This aligns with the findings of previous reports indicating that culture frequently fails due to factors, such as poor fungal viability, extensive tissue necrosis, or prior antifungal treatment [15–17]. While microscopy offers the advantage of rapid results, it is limited by its operator dependence and often fails to identify infections with low fungal burden [16].

The significantly higher sensitivity of PCR likely reflects its ability to detect fungal DNA from both viable and non-viable organisms. This feature provides critical diagnostic support, particularly in patients already undergoing antifungal therapy or in samples characterized by necrosis or degradation [18]. Findings of the present study are consistent with those reported by Tang et al. [19], who developed a Pan-Mucorales PCR assay followed by a multiplex species-specific PCR across 166 clinical specimens, demonstrating a Pan-Mucorales assay sensitivity of 98.6% and specificity of 100%. Their multiplex assay yielded species-level sensitivities ranging from 70.8% to 93.8% depending on the genus [19]. Compared with the in-house method developed by Tang, the MucorGenius® assay exhibited slightly lower sensitivity (76.5%) but equivalent specificity (100%). Differences in specimen diversity may partially explain this discrepancy, as our cohort included specimens, such as pus and pleural fluid, that tend to harbor lower fungal burdens and pose challenges in DNA extraction.

The relatively small sample size (n=27) in the present study may influence sensitivity estimates and restrict broader generalizability. Nevertheless, both studies support the clinical utility of real-time PCR in mucormycosis diagnosis, offering more rapid and sensitive detection than conventional approaches.

Complementary investigations reinforce the superiority of molecular diagnostics. For instance, Springer et al. demonstrated that a Mucorales-specific real-time PCR detected fungal DNA in 89% of proven cases, notably in histopathology-confirmed but culture-negative specimens [18]. Moreover, Guegan et al. reported 90% sensitivity and 97.9% specificity for the MucorGenius® assay in pulmonary samples, findings that closely mirror those observed in the present study despite differences in populations and specimen types [20]. Furthermore, Rafanomezantsoa et al. [21] compared three commercial assays-MycoGENIE, Fungiplex, and MucorGenius-and found MycoGENIE to have slightly higher sensitivity (83%) but noted its more limited genus coverage and higher cost, confirming that all assays maintain high specificity and perform best within comprehensive clinical algorithms.

The high PPV (100%) observed for the MucorGenius® assay in our cohort underscores its reliability in confirming mucormycosis infection and supporting early initiation of targeted antifungal therapy. This is of critical importance, given that even brief delays in amphotericin B treatment have been associated with significantly increased mortality [22, 23]. Moreover, the utility of molecular detection in less invasive specimens, such as BAL or pleural fluid, illustrates the potential of PCR to aid diagnosis when biopsy is contraindicated or impractical, particularly in hematology and post-transplant populations [24, 25].

Despite these advantages, there are several limitations. Currently, the MucorGenius® assay lacks detailed species-level resolution and does not provide antifungal susceptibility data, both of which are essential for tailoring antifungal treatment and monitoring shifts in epidemiology [26, 27]. Future efforts should aim to expand the genus coverage of the assay, develop multiplex molecular panels capable of detecting co-infections, and validate serum-based PCR methodologies that may enable preemptive screening in high-risk patients.

Ultimately, a multifaceted diagnostic approach that combines molecular PCR assays with histopathology, imaging, and validated clinical scoring systems-as advocated by recent guidelines from the European Confederation of Medical Mycology and the Mycoses Study Group Education and Research Consortium-represents the most reliable and timely strategy for diagnosing mucormycosis [28]. This integrated diagnostic framework offers the best prospect for improving clinical outcomes in this often-fatal infection.

## Conclusion

This study reaffirmed the superior performance of the MucorGenius® PCR assay, compared to traditional culture-based methods for the diagnosis of mucormycosis, particularly in culture-negative cases. The high specificity and rapid turnaround time of PCR enable early detection and timely intervention, both of which are critical for improving patient outcomes. High performance of the assay in detecting Mucorales DNA in various sample types, including tissue biopsies, BAL, and pleural fluid, underscores its utility in a wide range of clinical settings. Despite its advantages, the integration of PCR into routine clinical workflows requires overcoming challenges, such as the lack of antifungal susceptibility testing and the cost and availability of molecular diagnostics. Future advancements should focus on improving accessibility, sensitivity, and the combination of molecular diagnostics with immunological markers to further enhance the early detection and treatment of mucormycosis. Findings of this study support the inclusion of MucorGenius® PCR as part of a comprehensive diagnostic approach for mucormycosis, ensuring more accurate and timely diagnoses that could ultimately improve patient survival rates.
